# Framing ‘fracking’: Exploring public perceptions of hydraulic fracturing in the United Kingdom

**DOI:** 10.1177/0963662515595159

**Published:** 2015-07-13

**Authors:** Laurence Williams, Phil Macnaghten, Richard Davies, Sarah Curtis

**Affiliations:** University of Sussex, UK; Wageningen University, The Netherlands; Newcastle University, UK; Durham University, UK

**Keywords:** fracking, framing risk, lay expertise, participation in science policy, public engagement, risk perception, shale gas

## Abstract

The prospect of fracking in the United Kingdom has been accompanied by significant public unease. We outline how the policy debate is being framed by UK institutional actors, finding evidence of a dominant discourse in which the policy approach is defined through a deficit model of public understanding of science and in which a technical approach to feasibility and safety is deemed as sufficient grounds for good policymaking. Deploying a deliberative focus group methodology with lay publics across different sites in the north of England, we find that these institutional framings are poorly aligned with participants’ responses. We find that unease regularly overflows the focus on safety and feasibility and cannot be satisfactorily explained by a lack of understanding on the part of participants. We find that scholarship from science and technology studies productively elucidates our participants’ largely sceptical positions, and orientates strategies for responding to them more effectively.

## 1. Introduction

Hydraulic fracturing (commonly known as fracking) is a technique used for the extraction of oil or gas from ‘unconventional’ reservoirs such as shale. The technique involves drilling horizontally into layers of rock and injecting highly pressurised fracturing fluid (usually a blend of water, sand and various chemicals). Fracking was pioneered in the United States with shale gas rising from less than 1% of US domestic gas production in 2000 to over 20% in 2010 (Stevens, 2012), and with projected increases to nearly 50% by 2035 (US Energy Information Administration (US EIA, 2012). In the United Kingdom, the most recent resource estimates are 1329 trillion cubic feet (tcf) of gas-in-place for the Bowland shale, 80.3 tcf gas-in-place and 6 billion barrels (bbl) of shale oil for the Midland Valley of Scotland, and 4.4 bbl of shale oil in place for the Weald Basin (Andrews, 2013, 2014; Monaghan, 2014). These resource estimates refer to the total amount of resource present, as opposed to how much can technically and economically be expected to be produced (known as ‘reserves’ and typically smaller than resource estimates). Reliable UK reserve estimates are not currently possible due to a lack of understanding about the geology and a lack of experience of the engineering and costs of production (DECC, 2013a). Despite this, the technology has already been promoted as having strong potential to secure new energy supplies, promote economic growth and energy security, and assist in the transition away from carbon-intensive, coal-based energy (see, for example, Cameron, 2013; House of Lords Economic Affairs Committee, 2014).

However, fracking operations have, at the same time, generated controversy worldwide and have given rise to diverse and localised grassroots protest groups internationally. Environmental concerns have been raised about possible contamination of water resources (groundwater and surface water), high levels of water consumption and induced seismicity, alongside wider concerns about the industrialisation of rural landscapes, insufficiently regulated corporate power and community disempowerment (De Rijke, 2013; Steger and Milicevic, 2014; Wynveen, 2011). Securing public support for the development of unconventional oil and gas production is fast becoming a key strategic concern for UK and European policymakers (see, for example, Cameron, 2013; Obama, 2014).

There has been a growth in social science research on fracking recently, especially since 2010. While we do not offer a review here, it should be noted that this growing body of work has largely, though not exclusively, focused on three areas: namely, policy research (e.g. Rinfret et al., 2014); information and attitude surveys (e.g. Boudet et al., 2014); and fracking in the media (e.g. Jaspal and Nerlich, 2014). However, in a recent paper, Jaspal et al. (2014) claim that there is both a lack of research on the ‘public perceptions of fracking’ and consideration from a science and technology studies perspective (p. 502). This article aims to contribute to addressing both of these gaps. In particular, we explore the factors that are shaping the public controversy through qualitative research on public perceptions in the United Kingdom. We analyse both UK institutional framings of fracking policy and the understandings and framings of fracking articulated by lay participants (representing both latent and campaigning publics; see Mohr et al., 2013) derived from six in-depth qualitative focus groups held in early 2013. We then critique the institutional policy story-line using theory and concepts from debates in science and technology studies (STS), which we argue provide a set of conceptual resources that better recognise and accommodate these lay participant’s understandings, and which have implications for policymaking and further research.

## 2. Institutional framings of fracking

In 2013, the UK Office of Unconventional Gas and Oil (OUGO) was set up to oversee the development of unconventional energy resources. One of its core objectives is to ‘support public engagement’, described as ‘helping people understand the facts about unconventional gas and oil production and what it could mean if it takes place in their area’ (DECC, 2013b). In this regard, environmental risk assessments are proposed ‘to provide a full picture of the risks and impacts to inform effective engagement with local communities’ (DECC, 2012). Public and community engagement implies, according to this rhetoric, the provision to the public of established scientific knowledge about risks. The UK Prime Minister David Cameron adopted this rhetoric when he suggested that ‘[i]f neighbourhoods can see the benefits – and are reassured about its effects on the environment – then I don’t see why fracking shouldn’t receive real public support’ (Cameron, 2013). The Royal Society/Academy of Engineering (2012) study, set up at the request of the UK Government, reported that the ‘health, safety and environmental risks associated with hydraulic fracturing […] as a means to extract shale gas can be managed effectively in the UK as long as operational best practices are implemented and enforced through regulation’ (p. 4). This helped reinforce a policy story-line (see Hajer, 1996) in which the sole legitimate barriers to achieving ‘real public support’ are seen to be a failure on the part of the public to recognise the benefits of fracking and to be reassured by institutional commitments to effective risk assessment and management.

This institutional rhetoric contains echoes of a particular framing of the public understanding of science (PUS) built on a one-way model of science communication. According to this approach – known as the deficit model of science communication – it is assumed that public unease is caused primarily by a lack of sufficient knowledge (a deficit of understanding) and that the best way to overcome this is through the provision of accurate and didactic communication of scientific knowledge on risks and benefits, which will best engender public support and the acceptance of new technologies. The role prescribed for ‘local communities’ in processes of ‘participation’ and ‘engagement’ remains a largely passive one of receiving information, and where deliberation would be dominated by existing risk-science.

The dominant UK institutional framing of the debate on governance is that fracking should be permitted unless there is evidence of harms to the environment or human health provided through scientific environmental risk assessments. As with other technologies, this has been represented institutionally as the right and proper mode of governance (Kearnes et al., 2006; Wilsdon and Willis, 2004). After two small earthquakes were triggered at Preese Hall, Lancashire, in 2011, a ministerial statement by Edward Davey in December 2012 announced that ‘… appropriate controls are available to mitigate the risks of undesirable seismic activity’ and that ‘[o]n that basis, I am in principle prepared to consent to new fracking proposals for shale gas’ (DECC, 2012). This decision was presented as resting on scientific knowledge of risk. Questions and concerns beyond risk, or issues unrelated to safety, were deemed not to be sufficient grounds to influence the decision.

Institutional actors tend to assume that risk-science can determine whether institutionally defined thresholds of safety have been exceeded, and that this should constitute the legitimate basis for the determination of policy. However, this (inadvertently) is likely to downplay both other possible issues, which remain uncertain, and other modes of problem definition. For example, it remains a matter of debate as to how development of unconventional resources might be reconciled with climate change policies and targets (Broderick and Anderson, 2012; Broderick et al., 2011), especially with regard to timescale (McGlade et al., 2014), which hitherto have tended to be represented as too uncertain, particularly until reserve estimates are made, to frame policy deliberation (see, e.g., DECC, 2012). On the other hand, a 2011 House of Commons Energy and Climate Change Committee (ECCC) 2011 report concluded that:

there is no evidence that the hydraulic fracturing process itself poses a direct risk to underground water aquifers … hypothetical and unproven risk must be balanced against the energy security benefits that shale gas could provide to the UK … a moratorium in the UK is not justified or necessary at present. (p. 10)

Thus, issues that are largely beyond established risk-science knowledge are represented as insufficient grounds for informing policymaking, particularly in relation to calls for a moratorium, whereas the risks of seismicity and ground or surface water contamination are not deemed to be legitimate grounds for broader deliberation about the desirability of the technology itself (see RS/RAoE, 2012).

These commitments concerning what constitutes ‘good’ policy and the legitimate sites and modes of its making represent a common facet of the UK institutional story-line on fracking. Established scientific risk knowledge especially and technical knowledge about the potential benefits are presented as the legitimate sites and modes for the making of ‘good’ policy. ‘Good’ policy is viewed as resting on authoritative expertise, which will also be used to persuade and reassure resistant publics, and thus resolve controversy. Broader consideration of ‘hypothetical and unproven risk’ – of uncertainties and domains of ignorance – and broader deliberation around innovation choices and the social desirability of fracking are not viewed as justified or necessary. It is these assumptions that frame the research presented in this article, where we examine whether and how these policy narratives map onto lay publics’ understandings and framings of the fracking issue.

## 3. Methods

A deliberative focus group methodology was employed in the study, informed by examples of good practice and guidance on deliberative design (e.g. Davies et al., 2009). The methodology has been developed and applied to other analogous emerging technologies, including agricultural biotechnologies (Grove-White et al., 1997), nanotechnologies (Macnaghten, 2010) and solar radiation management (Macnaghten and Szerszynski, 2013), as a way of exploring latent public concerns at an early stage of a technology’s development.

Northern England in early 2013 was selected as a relevant setting for the research. At that time, the issues surrounding fracking were emerging in local and national debates although for most of the population it remained a rather novel issue. Following a pilot focus group in Durham, six deliberative focus groups of eight participants (a total sample of 48) were conducted with two groups organised in each of three locations – Newcastle, Nottingham and Lancashire (Chorley and Oldham). The groups were selected purposively as representing theoretically significant interests in the risks and benefits of fracking. Three groups shared a strong relationship with ‘the earth’ and practical interest in ‘the environment’: allotment holders in Newcastle, ex-miners in Nottingham and associates and employees of the Lancashire Wildlife Trust in Chorley, Lancashire. The remaining three groups shared a relevant positionality in relation to fracking as constituting a form of ‘progress’ through a strong relationship with time and the future. These groups were as follows: mothers with young children in Newcastle, members of local industrial history societies in Nottingham and parents of university students in Oldham, Lancashire. Selection criteria were based on the argument that diverse public groups can make well-informed contributions based on shared and topic-specific experiential knowledge (for an explanation of the idea of the theoretical sample, see Gobo, 2005). Further criteria used to ensure diversity of view within groups were age, gender and socioeconomic status, as set out in Table 1.

**Table 1.** Selection criteria used to recruit focus group participants.

Group	Name	Age	M/F	Class	Place	Topic-specific variable
1	Allotment holders	33–68	M/F	B–D	Newcastle	The earth (digging)
2	Mothers of young children	33–44	F	A–D	Newcastle	Time (the future)
3	Local history society	34–68	M/F	A–D	Nottingham	Time (the past)
4	Ex-miners	45–66	M	B–D	Nottingham	The earth (extraction)
5	Lancashire Wildlife Trust	22–67	M/F	A–D	Lancashire	The earth (environment)
6	Parents of university students	43–60	M/F	B–D	Lancashire	Time (the future)

Recruitment was ‘topic-blind’. Participants were told that they would take part in a discussion about issues relating to technology, energy and the environment. The groups were moderated by one of the authors and lasted 2 hours. A topic guide (list of questions and topics) and a series of concept boards (large A1 boards consisting of pictures, diagrams, newspaper headlines, quotes, etc.) were developed and refined through the pilot group. Material for the concept boards was selected and agreed by the authors to provide a coherent overview of the way fracking was being considered from various institutional perspectives. A moderating style was developed that aimed not to close down or narrow the issue, or presume that public understandings align with dominant institutional frames and norms. Table 2 details the topics of discussion and the stimulus materials.

**Table 2.** Stimulus material used in the focus groups and topics of discussion.

Topic	Concept board materials	Discussion themes
Introductory and contextual discussions	None	Personal relationship with energyProblems and benefits associated with energyEnergy in the mediaDifferent sources of energyDiscussion of priorities
Introducing fracking	Brief history of the fracking technique (source: Cuadrilla’s website)	Initial impressions and questions
	Brief technical description (source: Total’s website)	
	Brief description of the purpose of the process (source: DECC)	
	Diagrams of fracking fluid composition and the processes of fracking and horizontal drilling (source: Total’s website)	
Potential Benefits of Fracking	Description of increase in production in US gas production (source: US Energy Information Association)	Discussion of benefits, opportunities, and reasons for optimism.
	Prospect of a ‘golden age of gas’, including the role of unconventional sources (source: US Energy Information Association)	What it might mean personally and for society as a whole.
	Description of US price drops and potential economic benefits for Lancashire (source: Caudrilla’s website)	
	UK politicians Charles Hendry and George Osborne claiming benefits (source: media reports)	
	Headline of The Sunday Times, ‘The wonder gas that could cut your energy bills’	
	Front page of Time Magazine, ‘This rock could power the world’	
Potential risks of fracking	Scientific debate over the possibility of groundwater contaminationInformation about seismicity from DECC and the Tyndall CentreDebate on further possible impacts from the Tyndall CentreHeadline from The Times, ‘Fracking for shale gas caused Lancashire earthquakes, report’Headline from The New York Times ‘A tainted water well, and concern there may be more’Image of Gasland film ‘flaming tap’ scene.	Discussion of concerns on risks and of best strategy to adopt when faced with uncertainty. Discussion of potential benefits versus potential risks.

Audio recordings of each focus group, taken with participants’ informed consent, were professionally transcribed and then analysed by one of the authors. Analysis was conducted in accordance with agreed norms on qualitative data analysis (Barbour, 2007; Mason, 1996; Potter and Wetherell, 1987), through an iterative process of reading, thematic coding and reflection, in order to identify a series of key themes and sub-themes.

## 4. Results and analysis

In this section, we develop a thematic analysis of how people understood (1) the wider energy and society landscape, (2) the technique of fracking, (3) the potential benefits of fracking and (4) the potential risks of fracking.

### The energy and society landscape

At the start of each focus group, participants deliberated on a series of general questions about energy and society, designed to help elucidate the background context for subsequent responses to fracking. Although there was great diversity in these discussions, three themes emerged regularly and consistently across groups. The *affordability* of energy was a dominant theme, but with differences of emphasis. The affordability of energy was seen as the key priority for policymaking for the allotment holders (Group 1) and ex-miners (Group 4), while for the local history society members (Group 3) and for the wildlife trust employees (Group 5), concern was expressed that debates about energy were currently too focused on price, and that this was shifting the focus away from other and more important issues (such as the environment). For the latter groups (Groups 3 and 5), a strong emphasis was placed on the need to reduce consumption, to improve efficiency and to increase consumer power and choice.

*Industry behaviour* was a second theme that dominated early discussions. Participants across all groups displayed a heightened tendency to view industry actors as motivated by greed and profit, and that these motivations and associated practices would contribute to short-termism and negative impacts on society and the environment. For the mothers (Group 2), wildlife trust employees (Group 5) and parents (Group 6), a common theme was that powerful incumbent interests may be blocking and stifling innovation, or using their influence to promote environmentally destructive innovation pathways.

A third cross-cutting theme was concern over the prospects of *good governance* in relation to energy and climate change. This theme was particularly important to the wildlife trust employees (Group 5), who were concerned that we may have already left it too late to respond adequately to climate change, and who bemoaned the lack of a ‘coherent energy strategy’. The parents (Group 6) similarly emphasised a perceived lack of progress towards sustainable energy transitions, and lamented what they saw as a tendency to ‘constantly look for new fossil fuels’, which to them was short-sighted and irrational.

There were further areas of debate that were common but not shared across all groups. An issue that was specific to the ex-miners (Group 4) at this stage of the discussion was the claim that they had not as yet felt the claimed benefits that had been promised from North Sea Gas and Oil. For participants in the wildlife trust and parents groups (Groups 5 and 6), a common theme was that it was ‘human nature’ to put off difficult decisions until it was too late, and that we may need ‘a catastrophe’ to spur publics and policymakers into action. The local history society members (Group 3) and the parents (Group 6) articulated concerns about expertise of an epistemological and hermeneutic nature. The local history society members (Group 3), in a discussion of nuclear energy, emphasised the possibility of surprises that were likely to overflow the ability of experts and operators to know and control. They identified what sociologists of science call a ‘naïve sociology’ (Wynne, 1996; Yearley, 2005) on the part of experts in their apparent assumption that human behaviour (in relation to health and safety practices) would always and necessarily unfold in expected and unproblematic ways. The parents (Group 6) also noted this perceived naïve tendency of expert actors, and recognised their own troubling dependency on expertise, stating: ‘we go with what we’re told, don’t we’, it’s ‘the only option we’ve got’.

These early discussions, lasting on average 45 minutes, were designed to elicit a contextual understanding of how people are likely to respond to fracking and the factors deemed most probable to shape future public responses. What they illustrate is that various kinds of policy assumptions and conventional wisdom may be only partially captured in public sentiment: for example, that falling energy prices are always and necessarily desired at any perceived cost, or that the energy industry is always viewed as a benign creator of wealth. A further finding concerns ambivalence to what have been described as master and policy narratives of scientific governance (see Felt et al., 2007, on the notion of master narratives in European policymaking on science and technology). These ‘narratives’ refer to underlying descriptive and normatively performative collective imaginations that, in the context of science governance, both represent and shape the relations between science, technology and society (Felt et al., 2007). Even though participants commonly used arguments associated with these narratives – that there is a singular reality ‘out there’ that can be consummately known and controlled through the practices of science, that sound scientific knowledge is *the* legitimate basis for policymaking on issues with technical aspects, that scientific and technological innovation necessarily provides desirable outcomes and solutions or constitutes ‘progress’, and that innovation is a linear race with little scope or justification for social choice – this did not exhaust their discourse. Across each of the various groups, there were also challenges to these often taken-for-granted narratives. These challenges became more pronounced as discussions unfolded and will be discussed in greater detail below. A third and final finding was the sheer paucity of trust and goodwill extended towards industry and government. This is scarcely a novel revelation and is widely recognised as a key policy challenge. Again, the extent to which current institutional framings of fracking are likely to address or exacerbate this dynamic will be addressed below.

### The technique of fracking

Using the stimulus material discussed above, the participants then discussed the technique of fracking. For the allotment holders (Group 1) and the ex-miners (Group 4), the prospect of fracking was met with initial optimism. The ex-miners, in particular, set about trying to assess how many people would be employed on site, what sort of jobs would result, who they might go to, as well as posing technical questions concerning economic feasibility, the size of resource and the timescale of development. As debate progressed through the subsequent phases of the focus group, the ex-miners constantly returned to these sorts of questions, the answers to which (derived both from their interpretation of the stimulus materials and from wider experience) increasingly punctured this early sense of optimism.

The remaining four groups all started from a position of scepticism. The local history society members (Group 3) and the wildlife trust employees (Group 5) both focused on an epistemological debate about the likelihood of scientific disagreement, uncertainties and ignorance:

Dan: The impression is that there hasn’t been enough research done into this. Which makes you wonder just how dangerous it’s going to be?

(Focus group 5: Lancashire Wildlife Trust employees)

A persistent tendency among participants was to emphasise ‘what we don’t know’ over ‘what we do’, and to focus on the possibility of unforeseen consequences. Under the impression of incertitude, as above, participants were inclined to imagine a ‘worst-case scenario’. The local history group (Group 3) discussed the possible irreversibility of consequences, while drawing perceived parallels with the 2010 Deepwater Horizon oil spill in the Gulf of Mexico. The mothers (Group 2) and the wildlife trust employees (Group 5) both discussed fracking as a ‘short-term fix’, and wondered, therefore, whether it was the ‘right road to go down’. Finally, the mothers (Group 2) responded with disbelief and anger that they had not been informed about something that ‘seems like a massive thing to happen’:

Janet: Surely we as the people of the UK should have been informed that this was possibly going to start happening.

Marylin: Do they not give you a choice?

Emily: To me, this seems like a massive thing to happen.

Janet: To not have been …

Emily: I can’t actually believe I didn’t know.

(Focus group 2: Mothers of young children)

### The benefits of fracking

In response to the claimed benefits of fracking, depicted in the stimulus material as set out above, the two groups that had initially demonstrated optimism towards the technique of fracking articulated increasing scepticism as discussions about potential benefits matured. Both the allotment holders (Group 1) and the ex-miners (Group 4) found the idea that they would directly experience the benefits from fracking as highly dubious. These two groups generally agreed that affordability should be the most important priority for policymakers in initial discussions, but they were also the two groups for whom concerns over the trustworthiness of industry and policy actors were most pronounced:

Jason: Yeah, but [Moderator], do you really believe that our gas bills are going to go down? Because I just don’t believe it for one minute ….

Anthony: It’s a mistrust in politics and the government.

Darren: You can’t believe a word they say.

Anthony: Centrica’s your gas company, they’re going to sell it at the same price. They’re not going to go, ‘Oh, we’ll knock £50 off your gas bills’.

Darren: You’ll not get it cheaper. They’ll make a bigger profit on it.

Pete: But is it the companies or is it the government?

Darren: Everybody’s benefiting except the guy who’s having to pay his bill.

(Focus group 4: Ex-miners)

The initially sceptical groups were not won over by the claimed promises of benefits; instead, their positions arguably hardened. The mothers (Group 2) responded to the pronouncements of benefits as if they were ‘being sold something’, whereas the wildlife trust group (Group 5) dismissed the potential benefits as ‘hype’. The history society members (Group 3) completely refused to entertain the possibility of benefits, stating that:

Robert: It’s not really about that [the risks, the benefits], it’s about burning more fossil fuels and creating loads more CO_2_ …

Charlie: I question the actual end reason for it anyway, which is just more CO_2_ emissions. It’s just, like, you’re going down a road which is just a stupid thing to go down.

(Focus group 3: History society members)

These findings suggest that the potential benefits of fracking, as presented in the stimulus material, were far from self-evident for the overwhelming majority of the focus group participants. Some participants, especially the parents (Group 6) and the history society members (Group 3), were simply unwilling to consider the benefits of a technology designed to extend the development of fossil fuel resources. What the sceptical groups had in common was a focus on pathways, questioning whether fracking and unconventionals constituted a progressive, wise, path to pursue.

### The risks of fracking

Discussions in this section of the focus groups were frequently epistemological and hermeneutic in nature. Both the allotment holders (Group 1) and the parents (Group 6) responded to the expert claims (as presented in the stimulus material) that there were three possible mechanisms of water contamination by declaring that there very well could be more than three:

Paula: It can’t cover everything, I mean saying that these three things may happen, three possible ways of contamination, but that might not cover everything until something else happens along the line.

(Focus group 6: Parents of university students)

The mothers (Groups 2) and the ex-miners (Group 4) also shared a tendency to imagine, emphasise and take seriously ‘the worst-case scenario’, whether concerning possible water (ground or surface) contamination or induced seismicity. In addition, both the history society members (Group 3) and the parents (Group 6) argued that the claim made in the Royal Society/Royal Academy of Engineering (2012) report, that the risks of fracking are safely manageable assuming ‘operational best practices’ was an example of naive sociology, with the parents (Group 6) again making reference to the Deepwater Horizon oil spill as an analogy.

In response to the claim also made in the Royal Society/Royal Academy of Engineering (2012) report, that expected seismicity would likely be equal to or lower in magnitude than historical seismicity experienced in the UK due to coal mining, the mothers (Group 2) and history society members (Group 3) reflected that ‘social acceptability’ may well have shifted in the interim. The history society members (Group 3) rejected the argument that a precautionary approach may result in a missed opportunity by dismissing the argument as ‘just another way of selling it to us’, while the parents (Group 6) responded to the same point by suggesting ‘you don’t have to take an opportunity, do you? It’s a choice’. Finally the parents (Group 6) felt particularly strongly that exploratory drilling and fracking would constitute in practice a form of ‘testing’ and ‘experimenting’ with communities, with one participant suggesting she felt like ‘a guinea pig’. The perception shared by this group (all resident in Lancashire) was that the decision to commence exploratory activities in Lancashire had been determined with little consideration given to the communities and places that were implicated:

Alan: It just shows the values that they have, you know, they don’t value the residents very highly do they, so obviously they took into consideration that’s where they were going to get more oil or more fossil fuel out of that bit … They said, let’s do it here and sod the consequences.

(Focus group 6: Parents of university students)

Discussions in the ‘risk’ section of the focus groups were often dominated by epistemological, hermeneutic, axiological and democratic arguments. Many participants showed a tendency to question the story-line of dominant master and policy narratives as discussed above, and to resist the framing of the stimulus material which often tacitly embodied associated imaginations about science, technology and innovation. Claims of consummate knowledge and mastery were challenged; legitimate policy was seen not to rest solely on expert knowledge; fracking was seen frequently as a technology that could create more problems than solutions; and fracking was presented as simply one possible innovation trajectory that could be pursued more or less rigorously or possibly not at all. Moreover, the decision about whether to pursue fracking was widely seen as in danger of being rushed by policy and industry actors, and of being captured by powerful incumbent interests.

To summarise, in a number of important respects, the framing of the issues by lay publics were poorly aligned with current, dominant institutional framings of the issue. Although participant discourses were diverse, they can be organised thematically into four distinct but related key themes, considerably broader in scope than ‘safety and feasibility’, and poorly recognised, explained or addressed by the ‘deficit model’. First, participants questioned the *trustworthiness* of institutional actors and were reluctant to extend trust to industry or governance actors in the light of their experience of past interactions, presumed likely future behaviour and the sensed ambiguity over the purposes and intent driving decisions and commitments. This theme was particularly evident in participant’s sceptical wariness over promised benefits, and their distribution to the population at large. Second, participants expressed the importance of *inclusive* and democratic decision-making processes and sensed a lack of representation of the plurality of public issue-definitions and judgements. Expressions of alienation and dependency, concern over the possible capture of decision-making by powerful incumbent interests and the suspicion that deeply held views would be poorly represented in policy discourse all spoke to the importance of this theme. Third, participants expressed unease over the perceived *somnambulism* promoted by the restrictive ‘safety and feasibility’ institutional framing of the issue. Somnambulism – the condition of walking while asleep or in a hypnotic trance – is here employed as a metaphor to express the under-considered policy drift towards fracking perceived as underway by many participants, apparently unresponsive to public concerns and issue-definitions from beyond the ‘risk and feasibility’ framing. Appeals to the importance of choice, the focus on ‘pathways’, the need for a catastrophe and the representation of fracking as short-term fix, articulated the perceived need for more consideration in deciding on innovation trajectories, sensitive to public criticism and oriented to the long-term. Fourth, and finally, participants expressed a prevalent *epistemological pessimism* whereby uncertainty, ignorance and the ‘worst-case scenario’ were imagined and emphasised, and where experts tended to be characterised as naïve (in relation to assumptions about society) and complacent (in relation to an unruly, elusive nature). Interestingly, these themes map closely to the five spheres of concern (purposes, trust, inclusion, speed and equity) about the governance of science and technology identified by one of the authors in a meta-analysis of 17 UK public dialogue processes (Macnaghten and Chilvers, 2014).

## 5. Discussion

We now draw on scholarship from science and technology studies (STS) to sustain a critique of the institutional framings of fracking as presented above, to use this critique to help cast light on the largely sceptical positions expressed by our lay participants and to provide strategies for responding to them more effectively. The standard ‘risk communication’ model of technological governance has been systematically and routinely criticised by scholarship in the STS community, not least in research disseminated in this journal, for misrepresenting the diverse reasons that structure public concerns to potentially controversial science and technology. There is no guarantee that more information will lead to greater acceptance, or that the availability of facts will lead to a more ‘rational’ and calculative form of choice-making. Indeed, a wealth of studies have demonstrated that people use different kinds of information beyond ‘the risks’ to form judgements about a particular technology (see Sturgis and Allum, 2004 for a review of the literature). Other studies have developed a more radical critique, arguing that information about science cannot be separated from the institutional context in which it is produced, and thus that public perceptions of scientific information will depend, in part, on their experience of that institution’s behaviour (Wynne, 1996). This is seen as particularly important when these interactions are instrumental attempts to engender obedience (Wynne, 2006). Using this argument, Wynne suggests that science-based controversies are typically about expert and governing institutions who, through the dominance of scientific risk management in policy, ‘tacitly and furtively impose prescriptive models of the human and the social upon laypeople, and these are implicitly found wanting in human terms’ (Wynne, 1996: 57). He terms these issues, in the context of laypeople’s simultaneous relationship of dependency on these institutions and questioning of their trustworthiness, ‘social risks’ (Wynne, 1996).

The participants in our research articulated a great diversity of reasonable concerns over fracking, much of which cannot simply be put down to a failure to see the supposedly self-evident benefits or an irrational tendency not to be reassured by apparently compelling existing and established scientific risk knowledge. For example, concerns over *trustworthiness, inclusiveness* and *somnambulism* are obviously not reducible to known probabilities of adverse events. Even the regularly articulated *epistemological pessimism* is not the result of a deficit of understanding. Rather, our participants developed a story-line in stark contrast to the institutional policy framing on fracking and its reliance on a dominant set of assumptions and narratives about science, technology and innovation, instead emphasising the trustworthiness of institutions, the importance of inclusive and democratic decision-making, the need to avoid myopic somnambulism in innovation choices and a humble epistemology. Assuming our participants were not atypical, this research suggests that the policy question of the public acceptability of fracking must include the ability and willingness of governing institutions to recognise, encounter and accommodate diverse and polyvalent public views.

The underpinning of governance decision-making on scientific risk assessment is intended to render the policy debate ‘rational’ and free from the ambiguity and uncertainty of values, politics, economics, interests and other practicalities. However, this is problematic when it marginalises questions and concerns that come from beyond established risk-science. Mohr et al. (2013) suggest that good governance ‘requires a policy-process that is open to challenge and improvement from a broader range of inputs’ (p. i), but note the tendency to view policy involving science as a ‘special case’ which should be determined by scientific evidence. Wynne (2007) terms this tendency ‘science-protected politics’. This severely inhibits scope for institutional understanding of ‘public meaning’ and of alternative problem definitions as seen above, and relegates publics to the role of either ‘passive non-entities’ or ‘threats due to deficient understanding’ (Welsh and Wynne, 2013).

The *precautionary principle* counts the very possibility of a potential concern as the legitimate basis to open up questioning and scrutiny from a plural range of actors. For Stirling, precaution goes beyond questions of the state of scientific knowledge, constituting ‘a general discipline in technological choice’ (Stirling, 2010: 8). Stirling (2014) combines precaution with the concept of diversity, which together provides a strategy for moving towards ‘innovation democracy’ – an alternative approach to making ‘good’ policy. In this view, on issues like fracking, scientific risk knowledge alone is not sufficient grounds for making good policy, because in ‘novel, complex or rapidly changing’ circumstances ‘uncertainties cannot confidently be reduced to single definite probabilities’ and wider issues in innovation politics are left unaddressed (Stirling, 2014). Stirling advocates supplementing technical understandings of feasibility and safety with the strategies of precaution and diversity, in order to ‘urge a more rational consideration of different aspects of incertitude’, and ‘urge greater attention to alternatives’ – in terms of both alternative innovation pathways and a greater plurality of voices – respectively. In short, broader consideration of possible uncertainties and areas of ignorance, and broader, more plural, deliberation around innovation choices and the social desirability of fracking are not only justified and necessary, but rational too. For the participants in our research who were concerned with trustworthiness, inclusiveness, avoiding somnambulism, and who displayed a pessimistic epistemological outlook, a greater emphasis on precaution and diversity is a better-aligned policy approach than the current dominant focus on safety and feasibility.

## 6. Conclusion

We have argued that the fracking problem is not just about the existence of objective risks, nor just about the public’s ability to understand them, but also about the institutional ability and willingness to recognise, encounter and accommodate diverse and polyvalent public views, and that broader consideration of possible uncertainties and areas of ignorance, and broader, more plural, deliberation around innovation choices and the social desirability of fracking are justified, necessary and rational. We have also argued that reorienting the institutional approach to fracking in these ways will better respond to the themes of concern presented in this research: *trustworthiness, inclusiveness, somnambulism* and *epistemological pessimism*. Taken together, these themes of concern certainly problematise a restrictive ‘risk and feasibility’ framing of the issue, and in so doing call for more careful consideration of the role of institutional behaviour and unresponsive policymaking processes in the controversy. However, this point should not be taken to mean that public scepticism is limited to judgements about decision making and policy-making processes independently of what these decisions are about. There was regularly concern about the technology itself, its risks and other potential material implications (from global climate change to changes to the everyday lifestyle and character of local places) and the current state of knowledge about them. In the Dutch context, the Rathenau Instituut have called for a broadening and deepening of debate, including the need to ‘involve public opinions and criticism in policy development’ (Van Waes et al., 2014), which our findings echo. Early steps towards this broadening and deepening of debate in the UK context are emerging with a recent UK Government Sciencewise-funded set of public dialogue workshops reporting some issues similar to this paper, in particular the perception that decisions appeared to have been taken without a clear evidence base of risks and benefits, the perception that decisions were being taken without adequate public consultation, and a lack of confidence in decision-making bodies and their ability to act in the face of perceived vested interests (TNS BMRB, 2014).

Four key lessons for policymakers emerge from this research. First, it is important that policymakers avoid adopting the position of ‘salesperson’ for fracking, in other words of adopting the role that Pielke terms an ‘issue advocate’ (Pielke, 2007). ‘Salespeople’ in a position of partisan advocacy are not likely to be viewed as legitimate arbiters. It is worth reiterating that participants in this research frequently responded to current examples of policy discourse on fracking by suggesting that they felt they were being sold something. This is particularly important in the context of Wynne’s claim that publics are likely to be particularly resistant to emerging technology if their interactions with institutions are seen as instrumental and cynical attempts to engender obedience (Wynne, 2006). Second, it is important to submit the possible benefits of fracking (and thus the reasons to frack) to the same level of scrutiny as the risks (and thus some of the reasons not to frack). Participants were regularly weary of suspected ‘hype’ over benefits, and these judgements have arguably been subsequently vindicated to an extent (see recent comments from UKERC researchers Professor Jim Watson and Professor Mike Bradshaw in Harrabin, 2014). Third, it is necessary to avoid ‘post-political’ language. The sensed lack of a democratic voice in decision-making on fracking was a key concern for many of our participants. The Prime Minister David Cameron’s 2013 Sunday Times op-ed entitled ‘Why We Can’t Afford To Miss Out On Shale Gas’ is problematic in this context by implying, disingenuously, that actually there is no choice. As Stirling (2014) suggests, innovations are rarely ‘individually indispensable’ or ‘uniquely unacceptable’. Fourth, and finally, engagement must be a dialogue, not a monologue. As we have argued, technical assessments of feasibility and safety cannot monopolise the ‘public meaning’ of fracking, nor fully determine decisions on its trajectory. OUGO’s definition of public engagement discussed above is therefore problematic. Public engagement (invited or uninvited) is as much about policymakers learning about public issue definitions, competing visions of the future, and priorities, as it is about publics learning the facts. Verifying the efficacy of these recommendations in the case of fracking is at this stage difficult, and will require further inquiry and supportive findings. One potential type of situation in which these recommendations could be further scrutinised lies in designing future public engagement events that build on the aforementioned initial workshops conducted for OUGO/Sciencewise (TNS BMRB, 2014) and that use this (and related) research as a benchmark. To demonstrate efficacy, such initiatives must aim to replicate ‘best practice’ in dialogue practice, using criteria such as those specified by Michel Callon and colleagues, who place a particular emphasis on early and intense deliberation, on developing where possible shared definitions of the issues (including their political economy contexts), on ensuring diverse and broad participation (including those which do not represent established interests), on developing support structures that enable participants to develop mature and considered perspectives, and on the need for commitment to ongoing and longitudinal engagement (Callon et al., 2009).

Our findings are based on research limited by a small sample size, and are susceptible to criticisms concerning a lack of representativeness, and we do not claim this account to be exhaustive of public responses to this unfolding issue. An attitude survey has tracked the UK public’s attitudes to shale gas extraction since March 2012. These surveys charted a hardening of public opinion from August 2013, after the so-called Battle of Balcombe (where protests over exploratory drilling in the West Sussex village dramatically raised the profile of fracking in the UK national media and public consciousness; O’Hara et al., 2014). Our research (conducted prior to the ‘Battle of Balcombe’) has yielded, on the whole, a much more sceptical picture than the pre-Balcombe surveys. Our more deliberative methodology was arguably better-attuned to the extant potential for the hardening of public positions that was subsequently captured by survey research, and seems to suggest further scope for deepening resistance, alienation and, at best, sceptical tolerance. A combination of survey results and deeper, participatory research will arguably need to be triangulated to understand better unfolding public attitudes towards fracking and unconventionals. The ability to draw valid lessons from this research relies on the extent to which our participants are ‘typical’. Five out of the six groups were ‘latent publics’ (see Mohr et al., 2013) and therefore did not have strong pre-existing commitments on the issue (many were not even aware of fracking), and moreover two groups were initially optimistic about fracking. Although Group 5 (composed of wildlife trust employees) was a ‘campaigning public’ (see Mohr et al., 2013), and could therefore potentially be claimed to be impartial in some way, their concerns were nevertheless regularly shared by other latent groups.

Finally, scholarship from STS has been found to illuminate our participants’ responses and continuing public ambivalence towards fracking more generally. In particular, Wynne’s (1996) concept of ‘social risks’ is a useful explanatory resource in addressing the particular importance of hermeneutic judgements about incumbent institutions behaviour, and the apparent lack of reassurance of expert declarations of low risk, and Stirling’s (2014) programme for an ‘innovation democracy’ was found to be better aligned with participants’ views on innovation choice and their emphasis on uncertainties, ignorance and the ‘worst-case scenario’ than the risk-based model offered by the dominant institutional approach. We would argue, therefore, that further research needs to study the relationship between public understandings and institutional behaviour and decision-making processes, including the ability or willingness of governing institutions to recognise, encounter and accommodate multiple and diverse public values and meanings.

## References

[bibr1-0963662515595159] Andrews IJ (2013) The carboniferous Bowland Shale gas study: Geology and resource estimation. British Geological Survey for Department of Energy and Climate Change. Available at https://www.gov.uk/government/uploads/system/uploads/attachment_data/file/226874/BGS_DECC_BowlandShaleGasReport_MAIN_REPORT.pdf (accessed 11 September 2013).

[bibr2-0963662515595159] Andrews IJ (2014) The Jurassic shales of the Weald Basin: Geology and shale oil and shale gas resource estimation. British Geological Survey for Department of Energy and Climate Change. Available at: https://www.gov.uk/government/publications/bgs-weald-basin-jurassic-shale-reports (accessed 5 January 2015).

[bibr3-0963662515595159] Barbour R (2007) Doing Focus Groups. London: SAGE.

[bibr4-0963662515595159] Boudet H Clarke C Bugden D Maibach E Roser-Renouf C Leiserowitz A (2014) ‘Fracking’ controversy and communication: Using national survey data to understand public perceptions of hydraulic fracturing. Energy Policy 65: 57–67.

[bibr5-0963662515595159] Broderick J Anderson K (2012) Has US shale gas reduced CO_2_ emissions? Examining recent changes in emissions from the US power sector and traded fossil fuels. A report commissioned by The Co-operative and undertaken by researchers at the Tyndall Centre, University of Manchester Available at: http://tyndall.ac.uk/sites/default/files/broderick_and_anderson_2012_impact_of_shale_gas_on_us_energy_and_emissions.pdf (accessed 10 September 2013).

[bibr6-0963662515595159] Broderick J Anderson K Wood R Gilbert P Sharmina M Footitt A (2011) Shale gas: An updated assessment of environmental and climate change impacts. A report commissioned by The Co-operative and undertaken by researchers at the Tyndall Centre, University of Manchester Available at: http://www.tyndall.ac.uk/sites/default/files/coop_shale_gas_report_update_v3.10.pdf (accessed 10 September 2013).

[bibr7-0963662515595159] Callon M Lascoumes P Barthe Y (2009) On Acting in an Uncertain World: An Essay on Technological Democracy. Boston, MA: The MIT Press.

[bibr8-0963662515595159] Cameron D (2013) We cannot afford to miss out on shale gas. The Telegraph, 11 8 Available at: http://www.telegraph.co.uk/news/politics/10236664/We-cannot-afford-to-miss-out-on-shale-gas.html (accessed 7 May 2014).

[bibr9-0963662515595159] Davies S Macnaghten P Kearnes M (eds) (2009) Reconfiguring Responsibility: Deepening Debate on Nanotechnology. Durham, NC: Durham University.

[bibr10-0963662515595159] De Rijke K (2013) Hydraulically fractured: Unconventional gas and anthropology. Anthropology Today 29(2): 13–17.

[bibr11-0963662515595159] DECC (2012) Written ministerial statement by Edward Davey: Exploration for shale gas (Announcement), 13 12 Available at https://www.gov.uk/government/news/written-ministerial-statement-by-edward-davey-exploration-for-shale-gas (accessed 11 September 2013).

[bibr12-0963662515595159] DECC (2013a) Resources vs. reserves: What do estimates of shale gas mean? (Note), 27 7 Available at: https://www.gov.uk/government/publications/bowland-shale-gas-study (accessed 21 January 2015).

[bibr13-0963662515595159] DECC (2013b) New office to look at community benefits for shale gas projects (Press Release), 20 3 Available at: https://www.gov.uk/government/news/new-office-to-look-at-community-benefits-for-shale-gas-projects (accessed 11 September 2013).

[bibr14-0963662515595159] Energy and Climate Change Committee (ECCC) (2011) Shale gas – Fifth report of session 2010–12. Available at: http://www.publications.parliament.uk/pa/cm201012/cmselect/cmenergy/795/795.pdf (accessed 7 May 2014).

[bibr15-0963662515595159] Felt U Wynne B Callon M Gonçalves ME Jasanoff S Jepsen M (2007) Taking European knowledge society seriously. Report of the Expert Group on Science and Governance to the Science, Economy and Society Directorate, Directorate-General for Research, European Commission, Brussels Available at: http://ec.europa.eu/research/science-society/document_library/pdf_06/european-knowledge-society_en.pdf (accessed 9 May 2014).

[bibr16-0963662515595159] Gobo G (2005) Sampling, representativeness and generalizability. In: Gobo G Gubrium J Seale C Silverman D (eds) Qualitative Research Practice. London: SAGE, pp. 65–79.

[bibr17-0963662515595159] Grove-White R Macnaghten P Mayer S Wynne B (1997) Uncertain World: Genetically Modified Organisms, Food and Public Attitudes in Britain. Lancaster: Centre for the Study of Environmental Change, Lancaster University.

[bibr18-0963662515595159] Hajer M (1996) The Politics of Environmental Discourse. Oxford: Oxford University Press.

[bibr19-0963662515595159] Harrabin R (2014) Ministers’ shale gas ‘hype’ attacked. BBC News, 12 11 Available at: http://www.bbc.co.uk/news/uk-politics-30013668 (accessed 5 January 2015).

[bibr20-0963662515595159] House of Lords Economic Affairs Committee (2014) 3rd Report of Session 2013-2014 The Economic Impact on UK Energy Policy of Shale Gas and Oil. Available at: http://www.parliament.uk/business/committees/committees-a-z/lords-select/economic-affairs-committee/news/report-publication/ (accessed 2 July 2015).

[bibr21-0963662515595159] Jaspal R Nerlich B (2014) Fracking in the UK press: Threat dynamics in an unfolding debate. Public Understanding of Science 23(3): 348–363. 23942831 10.1177/0963662513498835

[bibr22-0963662515595159] Jaspal R Turner A Nerlich B (2014) Fracking on YouTube: Exploring risks, benefits and human values. Environmental Values 23(5): 501–527.

[bibr23-0963662515595159] Kearnes M Grove-White R Macnaghten P Wilsdon J Wynne B (2006) From bio to nano: Learning lessons from the UK agricultural biotechnology controversy. Science as Culture 15: 291–307.

[bibr24-0963662515595159] McGlade C Bradshaw M Anandarajah G Watson J Ekins P (2014) A bridge to a low-carbon future? Modelling the long-term global potential of natural gas – Research report, UKERC. Available at: http://www.ukerc.ac.uk/publications/gas-as-a-bridge.html (accessed 5 January 2015).

[bibr25-0963662515595159] Macnaghten P (2010) Researching technoscientific concerns in the making: Narrative structures, public responses and emerging nanotechnologies. Environment & Planning A 41: 23–37.

[bibr26-0963662515595159] Macnaghten P Chilvers J (2014) The future of science governance: Publics, policies, practices. Environment and Planning C: Government and Policy 32: 530–548.

[bibr27-0963662515595159] Macnaghten P Szerszynski B (2013) Living the global social experiment: An analysis of public discourse on geoengineering and its implications for governance. Global Environmental Change 23: 465–474.

[bibr28-0963662515595159] Mason J (1996) Qualitative Researching. London: SAGE.

[bibr29-0963662515595159] Mohr A Raman S Gibbs B (2013) Which publics? When? Exploring the policy potential of involving different publics in dialogue around science and technology. A report for Sciencewise. Available at: http://www.sciencewise-erc.org.uk/cms/which-publics-when/ (accessed 5 January 2015).

[bibr30-0963662515595159] Monaghan AA (2014) The carboniferous shales of the Midland Valley of Scotland: Geology and resource estimation. British Geological Survey for Department of Energy and Climate Change. Available at http://www.bgs.ac.uk/shalegas/ (accessed 5 January 2015).

[bibr31-0963662515595159] Obama B (2014) The state of the union address. Available at: http://www.whitehouse.gov/the-press-office/2014/01/28/president-barack-obamas-state-union-address (accessed 9 May 2014).

[bibr32-0963662515595159] O’Hara S Humphrey M Andersson J Jaspal R Nerlich B Knight W (2014) Public perception of shale gas extraction in the UK: Has balcombe bottomed out? Available at: http://www.scribd.com/doc/131787519/public-perceptions-of-shale-gas-in-the-UK-September-2014-pdf (accessed 5 January 2014).

[bibr33-0963662515595159] Pielke R (2007) The Honest Broker: Making Sense of Science in Policy and Politics. Cambridge: Cambridge University Press.

[bibr34-0963662515595159] Potter J Wetherell M (1987) Discourse and Social Psychology: Beyond Attitudes and Behaviour. London: SAGE.

[bibr35-0963662515595159] Rinfret S Cook J Pautz M (2014) Understanding state rulemaking processes: Developing fracking rules in Colorado, New York, and Ohio. Review of Policy Research 31(2): 88–104.

[bibr36-0963662515595159] Royal Society/Royal Academy of Engineering (2012) Shale gas extraction in the UK: A review of hydraulic fracturing. Royal Society and the Royal Academy of Engineering. Available at: http://royalsociety.org/uploadedFiles/Royal_Society_Content/policy/projects/shale-gas/2012-06-28-Shale-gas.pdf (accessed 10 September 2013).

[bibr37-0963662515595159] Steger T Milicevic M (2014) One global movement, many local voices: Discourse(s) of the global anti-fracking movement. Advances in Sustainability and Environmental Justice 15: 1–35.

[bibr38-0963662515595159] Stevens P (2012) The ‘Shale Gas Revolution’: Developments and changes. A report by Chatham House. Available at: http://www.chathamhouse.org/sites/default/files/public/Research/Energy,%20Environment%20and%20Development/bp0812_stevens.pdf (accessed 7 May 2014).

[bibr39-0963662515595159] Stirling A (2010) From enlightenment to enablement: Opening up choices for innovation. In: López-Claros A (ed.) The Innovation for Development Report. Palgrave Macmillan, pp. 199–210.Available at: http://www.sussex.ac.uk/Users/prfh0/stirling%20chapter%20in%20lopez%20claros%20on%20enablement.pdf (accessed 10 September 2013).

[bibr40-0963662515595159] Stirling A (2014) Making choices in the face of uncertainty: Strengthening innovation democracy. In: Annual Report of the Government Chief Scientific Adviser 2014. Innovation: Managing Risk, Not Avoiding It. Evidence and Case Studies, pp. 49–62. Available at: https://www.gov.uk/government/publications/innovation-managing-risk-not-avoiding-it (accessed 5 January 2015).

[bibr41-0963662515595159] Sturgis P Allum N (2004) Science in society: Re-evaluating the deficit model of public attitudes. Public Understanding of Science 13(1): 55–75.

[bibr42-0963662515595159] TNS BMRB (2014) A report on findings from public dialogue workshops. Available at: https://www.gov.uk/government/publications/public-engagement-with-shale-gas-and-oil (accessed 27 January 2015).

[bibr43-0963662515595159] US Energy Information Administration (US EIA) (2012) Annual Energy Outlook 2012. Available at: http://www.eia.gov/forecasts/aeo/pdf/0383(2012).pdf (accessed 7 May 2014).

[bibr44-0963662515595159] Van Waes A de Vries A van Est R Brom F (2014) Broadening the debate on shale gas – Guidelines for decision-making based on the Dutch experience. Rathenau Instituut Available at: http://www.rathenau.nl/en/publications/publication/broadening-the-debate-on-shale-gas.html (accessed 5 January 2015).

[bibr45-0963662515595159] Welsh I Wynne B (2013) Science, scientism and imaginaries of publics in the UK: Passive objects, incipient threats. Science as Culture 22: 540–566.

[bibr46-0963662515595159] Wilsdon J Willis R (2004) See-through Science: Why Public Engagement Needs to Move Upstream. London: Demos.

[bibr47-0963662515595159] Wynne B (1996) May the sheep safely graze? A reflexive view of the expert-lay knowledge divide. In: Lash S Szerszynski B Wynne B (eds) Risk, Environment and Modernity: Towards a New Ecology. Thousand Oaks, CA: SAGE, pp. 44–83.

[bibr48-0963662515595159] Wynne B (2006) Public engagement as a means of restoring public trust in science: Hitting the notes, but missing the music? Community Genetics 9: 211–220. 16741352 10.1159/000092659

[bibr49-0963662515595159] Wynne B (2007) Public participation in science and technology: Performing and obscuring a political-conceptual category mistake. East Asian Science, Technology & Society: An International Journal 1: 99–110.

[bibr50-0963662515595159] Wynveen B (2011) A thematic analysis of local respondents’ perceptions of Barnett Shale energy development. Journal of Rural Social Sciences 26: 8–31.

[bibr51-0963662515595159] Yearley S (2005) Making Sense of Science: Understanding the Social Study of Science. London: SAGE.

